# Altered diversity and composition of gut microbiota in Korean children with food allergy

**DOI:** 10.1002/clt2.70036

**Published:** 2025-03-12

**Authors:** Minyoung Jung, Ji Young Lee, Sukyung Kim, Jeongmin Song, Sehun Jang, Sanghee Shin, Min Hee Lee, Mi Jin Kim, Jiwon Kim, Han Byul Lee, Yeonghee Kim, Kangmo Ahn, Minji Kim, Jihyun Kim

**Affiliations:** ^1^ Department of Pediatrics Samsung Medical Center Sungkyunkwan University School of Medicine Seoul South Korea; ^2^ Department of Pediatrics Hallym University Chuncheon Sacred Heart Hospital Chuncheon South Korea; ^3^ Department of Pediatrics Chung‐Ang University Hospital Chung‐Ang University College of Medicine Seoul South Korea; ^4^ Department of Pediatrics Chungnam National University Sejong Hospital Sejong South Korea; ^5^ Research Institute for Future Medical Science Chungnam National University Sejong Hospital Sejong South Korea; ^6^ Department of Health Sciences and Technology Samsung Advanced Institute for Health Sciences & Technology Seoul South Korea

**Keywords:** 16S rDNA sequencing, diversity, food allergy, microbiome, richness

## Abstract

**Background:**

This study aimed to comprehensively characterize the gut microbiome and identify individual and grouped gut microbes associated with food allergy (FA) using 16S rRNA gene sequencing.

**Methods:**

Fecal samples were collected from children with IgE‐mediated FA and from sex‐ and age‐matched controls. The V3–V4 variable regions of the 16S rRNA gene of the gut microbiome were profiled using next‐generation sequencing (Illumina, USA). Bacterial species richness, intracommunity diversity, and intergroup dissimilarity were evaluated. Functional profiles were predicted using Phylogenetic Investigation of Communities by Reconstruction of Unobserved States (PICRUSt) and the Minimal Set of Pathways (MinPath) algorithm.

**Results:**

Fecal samples were collected from children with IgE‐mediated FA (*n* = 66) and from sex‐ and age‐matched controls (*n* = 22). Gut microbiome richness (*p* < 0.0001), intra‐community diversity (*p* < 0.0001), and inter‐community diversity (*p* = 0.0004) were higher in the healthy group than in the FA group. Patients with FA were enriched in *Blautia*, *Fusicatenibacter*, and *Ruminococcus_g5* compared with healthy control individuals (all *p* < 0.05). Healthy control individuals were significantly enriched in *Oscillibacter* and *Ruminococcus* compared with patients with FA (all *p* < 0.05). Functional pathway analysis identified enrichment in pathways related to endoglucanase in healthy controls and the ATP‐binding cassette (ABC) transport system in FA patients.

**Conclusions:**

The gut microbiomes of patients with FA and healthy control individuals had different taxonomic abundances, and the microbiome richness and diversity of the bacterial flora of patients with FA were reduced compared with controls.

## 1 INTRODUCTION

Food allergy (FA), predominantly observed in the pediatric population, is attributed to the immaturity of the gut mucosal barrier and immune system.^1^ Recent evidence has highlighted the pivotal role of the microbiome in human health, particularly through modulation of mucosal barrier integrity, metabolism, and immune responses.^2^, ^3^ The interactions between the gut microbiota and the host's innate and adaptive immune systems within the gut are essential for inducing tolerance to food proteins.^4^, ^5^ The establishment and functional development of the gut microbiome during infancy are critical, involving mechanisms such as the induction of regulatory T (Treg) cells by short‐chain fatty acids and the shaping of innate immunity by microbiota‐derived *lipopolysaccharides*.^6^

The composition of the gut microbiome influences the spectrum of microbially derived metabolites, including both pro‐inflammatory and anti‐inflammatory agents.^7^ Commensal bacteria competitively inhibit the colonization of pathogenic bacteria and the production of metabolically defensive compounds.^8^ Notably, the presence of *Prevotella copri* at 6 months of age has been linked with a reduced FA incidence at 1 year of age.^9^ Conversely, a lower abundance of the genus *Clostridium* before 6 months has been observed in infants with allergic sensitization, though the class Clostridia has been reported to facilitate tolerance in children with cow's milk allergy.^10^, ^11^ This is further supported by findings that a decrease in Clostridiales populations in infants with FA suggests that fecal microbial transplantation of these species could prevent FA by promoting Treg cells in mice.^12^ Our recent cohort analysis also revealed a subgroup with food tolerance characterized by higher levels of beneficial bacteria such as *Prevotella*, *Lachnospiraceae*, and *Clostridium*.^13^

Despite these insights, direct comparisons of gut microbiota composition between children with FA and healthy controls have been limited, although the gut microbiome depends on diet, habits, and ethnicity.^14^ An in‐depth analysis of gut microbiota diversity in FA patients, compared with well‐matched controls, revealed microbiome signatures specific to different FA types.^15^ In this study, we aimed to investigate differences in gut microbiome composition between age‐ and sex‐matched healthy controls and FA patients to offer a comprehensive perspective on the gut microbiota and its association with FA in children.

## 2 METHODS

### 2.1 Study population

We enrolled 66 patients with FA and compared them to 22 age‐ and sex‐matched healthy controls between June 2021 and December 2022. The diagnosis of FA was based on a positive oral food challenge and serum specific immunoglobulin E levels ≥ 0.35 kU/L, measured using ImmunoCAP (Thermo Fisher Scientific Inc., Waltham, MA, USA). The healthy control group consisted of children who had never been diagnosed with allergic diseases such as atopic dermatitis, allergic rhinitis, asthma, and FA. We collected demographic data, medical history, family history of allergic diseases, and allergic reactions to the offending foods. This study was approved by the Institutional Review Boards of Samsung Medical Center (SMC) and Kosin University Gospel Hospital (KUGH) (IRB numbers: SMC 2020‐06‐124 and KUGH 2020‐11‐005), and written informed consent was obtained from all parents and/or patients prior to participation in this study.

### 2.2 Fecal sample collection, genomic DNA extraction, and bacterial 16S rRNA sequencing

The participants were provided with an OMNIgene‐GUT collection kit (OMR‐200; DNA Genotek, Ottawa, Canada), which included a protocol for self‐collection of stool samples. The study participants did not have any antibiotic exposure in the 7 days prior to collection. The collected fecal samples were stored at −80°C. Total DNA was extracted using the FastDNA Spin kit (MP Biomedicals, Irvine, CA, USA) or a Maxwell RSC PureFood GMO and Authentication Kit (Promega, Madison, WI, USA) according to the manufacturer's instructions. Polymerase Chain Reaction (PCR) amplification was performed using fusion primers targeting the V3–V4 regions of the 16S rRNA gene with the extracted DNA. The amplifications were carried out under the following conditions: initial denaturation at 95°C for 3 min, followed by 25 cycles of denaturation at 95°C for 30 s, primer annealing at 55°C for 30 s, and extension at 72°C for 30 s, with a final elongation at 72°C for 5 min. PCR products were confirmed by 1% agarose gel electrophoresis and visualized using a Gel Doc system (BioRad, Hercules, CA, USA). Amplified products were purified using CleanPCR (CleanNA, Waddinxveen, Netherlands). Equal concentrations of purified products were pooled, and short fragments (non‐target products) were removed using CleanPCR (CleanNA). The quality and product size were assessed using a Bioanalyzer 2100 (Agilent, Palo Alto, CA, USA) with a DNA 7500 chip. Mixed amplicons were pooled, and sequencing was carried out at CJ Bioscience, Inc. (Seoul, Korea) using an Illumina MiSeq Sequencing system (Illumina, USA) according to the manufacturer's instructions. Data analyses were performed using the EzBioCloud database (http://www.ezbiocloud.net).

### 2.3 Statistical analysis

Data were analyzed using Statistical Package for the Social Sciences (SPSS) for Windows (version 27.0; SPSS, Chicago, IL, USA) and Graphpad Prism (version 9.00; Graphpad Software, San Diego, CA, USA). Chi‐square test and Fisher's exact test were used to determine the differences in proportions. The Mann–Whitney *U* test was used to identify statistically significant pairwise differences between groups. The Student's *t*‐test was used to test the association with alpha diversity. Chao1 and operational taxonomic units (OTUs) were used to determine the richness of the samples. The alpha diversity calculated using the Shannon diversity and Simpson indices was determined using phylogenetic distance and the detected number of species metrics. Microbial beta diversity was determined using the generalized weighted UniFrac distance test at the OTU level to determine the differences in bacterial communities among the groups. The association between microbiome composition and covariates was tested using PERMANOVA. Linear Discriminant Analysis (LDA) with effect size estimation (LEfSe) scores was used to measure the consistency of differences in relative abundance between the control and FA groups. LDA effect size > 4.0 (among OTUs with > 1% relative abundance in any group) was used to distinguish significant differences in abundance of OTUs between control and patients with FA. All OTU tables and OTU taxonomies were mapped onto the Kyoto Encyclopedia of Genes and Genomes (KEGG) pathways. Using functional profiles by Phylogenetic Investigation of Communities by Reconstruction of Unobserved States (PICRUSt) and Minimal Set of Pathways (MinPath) algorithms, taxonomic biomarkers and functional biomarkers were discovered using statistical comparison algorithms (LEfSe and Kruskal‐Wallis H test).^16^, ^17^

## 3 RESULTS

### 3.1 Study population

We collected fecal samples from 66 children with FA [median, interquartile range] (4.0, 3.0–5.0 years) and 22 healthy children (4.0, 3.0–5.0) (Table S1). There were no significant differences in age (*p* = 0.911) or sex (*p* = 0.069) between the FA and control groups. Hen's egg (27/66) was the most frequent food allergen, followed by cow's milk (20/66) and wheat (19/66). Of the 66 patients with FA, 20 (30.3%) had a history of anaphylaxis (Table S1).

### 3.2 Comparison of richness and diversity indices

**FIGURE 1:**
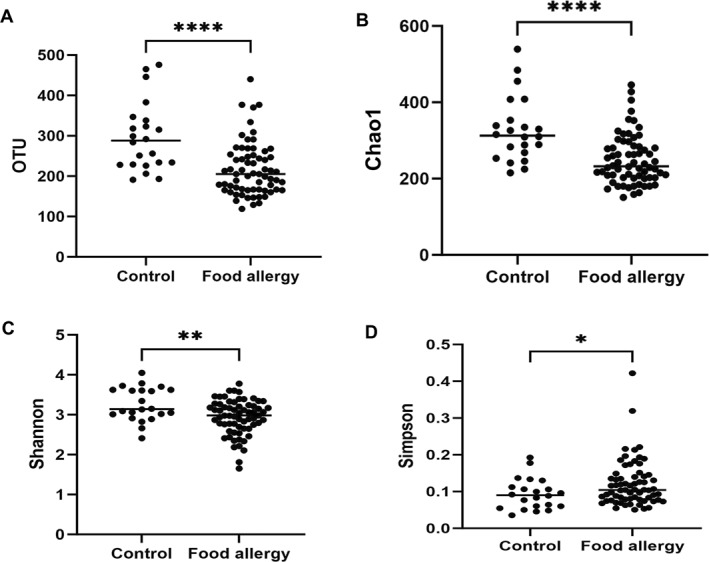
Comparison of gut microbiome richness and alpha diversity between control and food allergic patients. (A) OTU level, (B) Chao1 index, (C) Shannon index, and (D) Simpson index. *p < 0.05, **p < 0.01, ***p < 0.001, and ****p < 0.0001.

**FIGURE 2:**
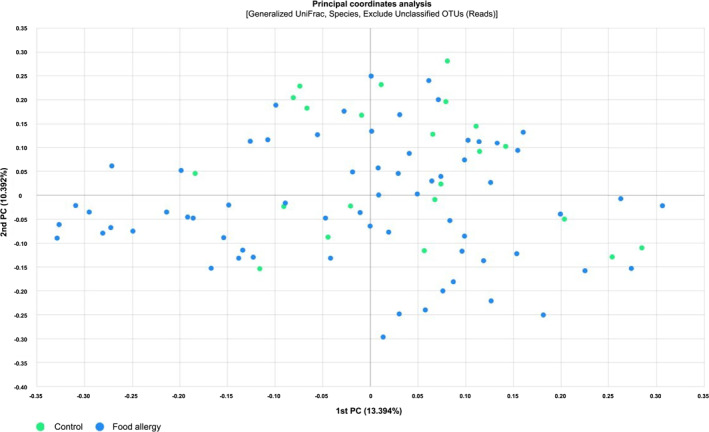
The principal coordinate analysis plot provides a visual depiction of similarity and dissimilarity between controls and patients with food allergy.

The number of OTUs in samples from the control and FA groups significantly differed in the median values, with 288.0 (IQR: 228.8–340.3) and 205.0 (IQR: 166.5–252.5), respectively (*p* < 0.0001) (Figure 1A). Additionally, the Chao1 index, another measure of species richness, also showed significant differences between the control (median: 312.9, IQR: 264.4–366.9) and FA groups (median: 232.3, IQR: 202.5–284.3, *p* < 0.0001) (Figure 1B). No significant differences were observed in the diversity of the three specific food types (OTUs; *p* = 0.317, Chao1; *p* = 0.633). Alpha diversity, which refers to intra‐community diversity, was measured based on the Shannon and Simpson indices. The median Shannon diversity index was 3.14 (IQR: 2.98–3.62) for the control group and 2.98 (IQR: 2.68–3.25) for the FA group (*p* = 0.0059) (Figure 1C). The Simpson index also showed significant differences, with median values of 0.09 for the control group and 0.10 for the FA group (*p* = 0.0421) (Figure 1D). No significant differences in diversity among the three food types were observed (*p* = 0.951 for Shannon and *p* = 0.836 for Simpson). To determine the degree of inter‐group dissimilarity, beta diversity was analyzed using generalized weighted UniFrac distance matrices (Figure 2). Beta diversity revealed significant differences between the control and FA groups (*p* = 0.0004).

### 3.3 Taxonomic composition and relative abundance analysis

**FIGURE 3:**
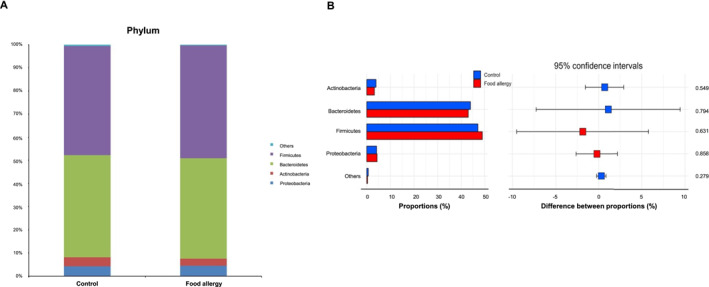
Bacterial relative abundance between controls and patients with food allergies. (A) Relative abundance at the phylum level and (B) gut microbiota composition at the phylum level.

The analysis revealed the top four phyla in relative abundance among all samples, with further detail provided for 35 genera. Bacterial taxa identified at an average abundance of less than 1% were classified as others. The dominant bacterial phyla in the control group were Firmicutes (47.2%), Bacteroidetes (44.1%), Proteobacteria (4.2%), and Actinobacteria (3.9%) (Figure 3A). In the FA group, the main phyla were Firmicutes (48.8%) followed by Bacteroidetes (43.3%), Proteobacteria (4.4%), and Actinobacteria (3.2%). No statistically significant differences were found between the groups at the phylum level (Figure 3B).

**FIGURE 4:**
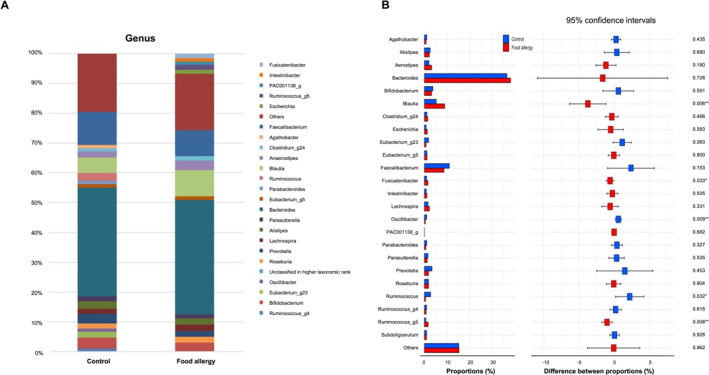
Bacterial relative abundance between controls and patients with food allergies. (A) Relative abundance at the genus level and (B) gut microbiota composition at the genus level.

At the genus level, the predominant taxa in the control group were *Bacteroides* (36.4%), *Faecalibacterium* (11.0%), *Blautia* (5.2%), *Bifidobacterium* (3.7%), *Prevotella* (3.4%) and *Ruminococcus* (2.8%). The main genera in the FA group were *Bacteroides* (38.4%), followed by *Faecalibacterium* (8.6%), *Anaerostipes* (3.2%), *Bifidobacterium* (3.1%), *Lachnospira* (2.2%), and *Alistipes* (2.2%) (Figure 4A). At the genus level, the abundance of *Blautia*, *Fusicatenibacter*, and *Ruminococcus_g5* in the FA group was significantly higher than that in the control group (*p =* 0.006, 0.033, and 0.008, respectively) (Figure 4B). In addition, the abundance of *Ruminococcus* and *Oscillibacter* was significantly higher in the control group than in the FA group (*p* = 0.032 and 0.009).

**FIGURE 5:**
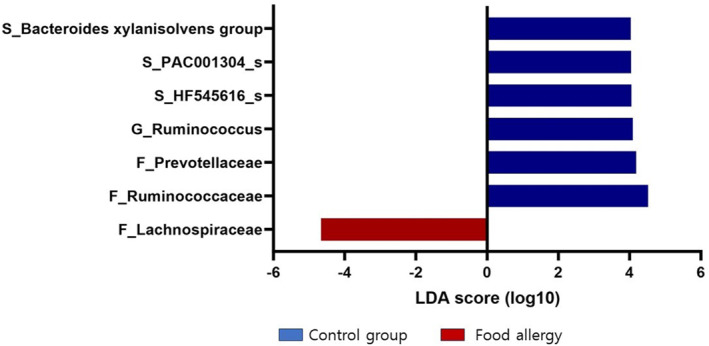
Taxonomic composition of gut microbiota in patients with food allergy. Only taxa with an LDA score more than 4 and *p* < 0.05 in the Wilcoxon signed‐rank test are shown. LDA = Linear discriminant analysis.

LEfSe analysis identified significant differences in the abundance of OTUs between controls and patients with FA using the threshold values (LDA > 4.0, *p* < 0.05). At the family level, *Ruminococcaceae* and *Prevotellaceae* were more abundant in the control group than in the FA group, whereas *Lachnospiraceae* was more abundant in the FA group. At the genus level, *Ruminococcus* was more abundant in the control group than in the FA group (Figure 5).

### 3.4 Predicted functional pathway of gut microbial taxa associated with food allergy

**FIGURE 6:**
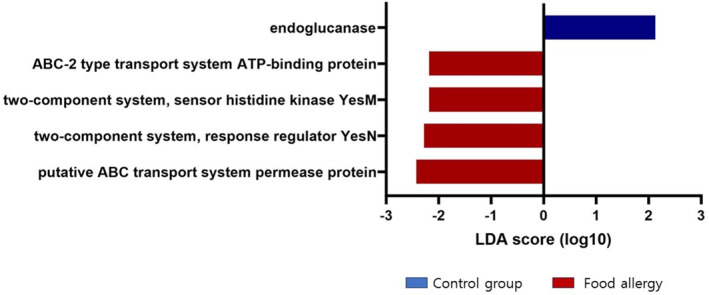
LEfSe analysis revealed distinct KEGG pathway differences in gut microbiota between controls and patients with food allergy. The KEGG pathways enriched in patients with food allergy are shown in red, while those enriched in control children are shown in blue. Only the taxa with a significant LDA score > 2 are displayed. KEGG = Kyoto Encyclopedia of Genes and Genomes, LDA = Linear discriminant analysis, LEfSe = Linear discriminant analysis effect size.

LEfSe revealed distinct KEGG pathway differences between the gut microbiome of children with FA and that of healthy controls using the threshold values (LDA > 2.0, *p* < 0.05) (Figure 6). Specifically, the pathway involving endoglucanase was enriched in the healthy control group compared with the FA group. In contrast, several pathways were more prominent in the FA group, including those associated with the putative ATP‐binding cassette (ABC) transport system permease protein, the two‐component system response regulator YesN, the sensor histidine kinase YesM, and the ABC‐2 type transport system ATP‐binding protein.

## 4 DISCUSSION

There is growing evidence that alterations in the gut microbiome are associated with FA, although the specific microbes involved in the pathogenesis of FA remain unknown. Our study used 16S rRNA gene sequencing to identify the differences in gut microbial diversity and the presence of individual microbes between children with FA and the healthy group. We found that patients with FA had a higher prevalence of specific microbes such as *Blautia*, *Fusicatenibacter*, and *Ruminococcus_g5*, whereas healthy individuals were significantly enriched in *Oscillibacter* and *Ruminococcus*. In addition, our findings indicate that the richness of the gut microbiome and both intra‐ and inter‐community diversity were significantly elevated in the healthy group compared with the FA group.

Microbiome colonization in infants is typically complete within the first month of life, and it undergoes significant changes with weaning and the introduction of solid foods.^18^ An Australian birth cohort study employing a microbiota‐by‐age Z score identified delayed maturation of the infant gut microbiota at 1 year to be associated with an elevated risk of IgE‐mediated FA at the same age.^9^ The first 2 years of life are pivotal for the interaction of the infant gut microbiome with the immune system.^13^, ^18^ Despite several studies on FA and the gut microbiome, findings have been heterogeneous, focusing on microbiome profiles during infancy or the development of FA after infancy, or have been collected from a limited number of patients.^11^, ^19^, ^20^ Some studies also included patients with non‐IgE‐mediated FA, diverging from the focus on immediate IgE‐mediated FA, resulting in mixed outcomes based on specimen collection timing and patient demographics.^21^ This underscores the need for data from well‐designed studies.

Previous studies have reported conflicting results on the distribution of gut microbiota between FA patients and controls.^22^ A U.S. study involving 141 children with egg allergy identified an abundance of *Lachnospiraceae*, *Streptococcaceae,* and *Leuconostocaceae* in the gut microbiota compared to controls.^23^ Conversely, an Italian study on 39 children with milk allergy found a higher diversity index dominated by *Lachnospiraceae* and *Ruminococcaceae* among FA patients. In contrast, our study identified a specific dominant microbiome composition of *Blautia*, *Fusicatenibacter*, and *Ruminococcus_g5* in FA patients, with a reduced presence of *Ruminococcus* and *Oscillibacter* compared with healthy controls. In our study, *Lachnospiraceae* including *Blautia* and *Fusicatenibacter* was more prevalent in the FA group.^24^ Although not statistically significant, our results suggest a trend toward a greater abundance of Bacteroidetes in the control group and Firmicutes in the FA group. Similar results have been observed in animal models, where sensitization with a food allergen led to an increase in the abundance of Firmicutes and its families such as *Lachnospiraceae* and *Erysipelotrichaceae*.^25^ Further analysis showed that a food sensitization group exhibited lower diversity, decreased Bacteroidetes, and increased Firmicutes compared to a control group.^20^ Previous studies either did not compare the diversity of the gut microbiota or reported no significant differences in diversity.^15^, ^19^, ^21^ The present study showed greater richness and diversity in the control group than in the FA group, while no differences were observed in diversity among the egg, milk, and wheat FA groups.

PICRUSt is a computational method for predicting the functional structure of a metagenome, and it may suggest a protective microbial function against FA.^17^ Endoglucanase, an enzyme that hydrolyzes large polysaccharides, interacts with a variety of immune receptors, including Dectin‐1, complement receptor, and Toll‐like receptor, resulting in the activation of dendritic cells, macrophages, neutrophils, monocytes, and natural killer cells.^26^, ^27^, ^28^ Despite limited evidence of its presence in the human gut, it is plausible that gut microbiota might express this function, potentially influencing dietary fiber breakdown and the generation of short‐chain fatty acids, which are beneficial for gut health and immune regulation.^29^ In addition, ABC transporters, known for their protective barrier functions and involvement in inflammatory mechanisms, were highlighted.^30^, ^31^ Interestingly, the participation of *Lachnospiraceae* in the ABC transporter metabolic pathway, as seen in animal studies, underscores the complex interactions among diet, microbial metabolism using butyric acid, and immune responses.^32^ However, these functional predictions are not direct measurements, which limits the interpretability of the results.

This study has several limitations, including its cross‐sectional design, which precludes the establishment of causal relationships. Additionally, by focusing solely on the microbiome, it does not fully explore other aspects of the gut environment that interact with bacterial flora. Despite these limitations, our findings could elucidate functional microbial signatures associated with FA, contributing to the identification of microbial biomarkers and the development of new therapeutic strategies. In particular, our insights into the gut microbiome's composition and functional potential may guide the optimization of fecal microbiota transplantation (FMT) and oral immunotherapy (OIT) with probiotics for FA. The pivotal role of the gut microbiome in the development of allergic diseases has increased interest in therapeutic interventions such as FMT and oral probiotics, especially in the context of OIT for peanut allergy.^33^, ^34^, ^35^ In the long term, our study's contributions to understanding the role of the gut microbiome in FA could lead to more effective microbiome‐based therapeutic strategies.

In conclusion, the results of this study showed a higher relative abundance of *Blautia*, *Fusicatenibacter,* and *Ruminococcus_g5* in children with IgE‐mediated FA and lower gut microbiome diversity and richness in FA patients than in controls. These associations with specific bacterial lineages require further investigation to clarify their roles in FA.

## AUTHOR CONTRIBUTIONS

**Minyoung Jung:** Investigation; conceptualization; methodology; writing–original draft; writing–review and editing; data curation; formal analysis. **Ji Young Lee:** Writing–review and editing; visualization; formal analysis; data curation; investigation. **Sukyung Kim:** Writing–review and editing; visualization; formal analysis; data curation; investigation. **Jeongmin Song:** Writing–review and editing; visualization; formal analysis; data curation; investigation. **Sehun Jang:** Writing–review and editing; visualization; formal analysis; data curation; investigation. **Sanghee Shin:** Writing–review and editing; visualization; formal analysis; data curation; investigation. **Min Hee Lee:** Visualization; data curation; investigation. **Mi Jin Kim:** Writing–review and editing; visualization; formal analysis; data curation; investigation. **Jiwon Kim:** Writing–review and editing; visualization; formal analysis; data curation; **Han Byul Lee:** Visualization; formal analysis; data curation; investigation. **Yeonghee Kim:** Visualization; data curation; investigation. **Kangmo Ahn:** Investigation; conceptualization; writing–review and editing. **Minji Kim**: Funding acquisition; investigation; conceptualization; writing–review and editing; formal analysis; supervision; resources. **Jihyun Kim:** Funding acquisition; funding acquisition; investigation; conceptualization; writing–review and editing; formal analysis; supervision; resources.

## CONFLICT OF INTEREST STATEMENT

The authors declare no conflicts of interest.

## Supporting information

Supporting Information S1

Table S1

Figure S1
