# Supplementary material for: Altered diversity and composition of gut microbiota in Korean children with food allergy
Source: Clin Transl Allergy. 2025 Mar 12;15(3):e70036. doi: 10.1002/clt2.70036 (PMC11903216; doi:10.1002/clt2.70036)
Supplement: Supplementary file 1 — Supporting Information S1 [file CLT2-15-e70036-s003.docx]

**Figure S1. Comparison of gut microbiome characteristics between children with food allergy and healthy controls.**
