# Supplementary material for: Altered diversity and composition of gut microbiota in Korean children with food allergy
Source: Clin Transl Allergy. 2025 Mar 12;15(3):e70036. doi: 10.1002/clt2.70036 (PMC11903216; doi:10.1002/clt2.70036)
Supplement: Supplementary file 2 — Table S1 [file CLT2-15-e70036-s001.docx]

**Table S1.** Participant demographics and baseline characteristics

|  | Control (n = 22) | Food allergy (n = 66) | *P*-value |
| --- | --- | --- | --- |
| Sex (male) | 11 (50.0) | 47 (71.2) | 0.069 |
| Age (yr) | 4.0 (3.0-5.0) | 4.0 (3.0-5.0) | 0.911 |
| Allergy history |  |  |  |
| Atopic dermatitis | 0 (0) | 38 (57.6) | 0.000002 |
| Asthma | 0 (0) | 10 (15.2) | 0.052 |
| Allergic rhinitis | 0 (0) | 23 (34.8) | 0.001 |
| Allergic family history | 0 (0) | 34 (51.5) | 0.000017 |

Values are median (interquartile range) or number (percentage).
