# Supplementary material for: Altered diversity and composition of gut microbiota in Korean children with food allergy
Source: Clin Transl Allergy. 2025 Mar 12;15(3):e70036. doi: 10.1002/clt2.70036 (PMC11903216; doi:10.1002/clt2.70036)
Supplement: Supplementary file 3 — Figure S1 [file CLT2-15-e70036-s002.pptx]

## Slide 1
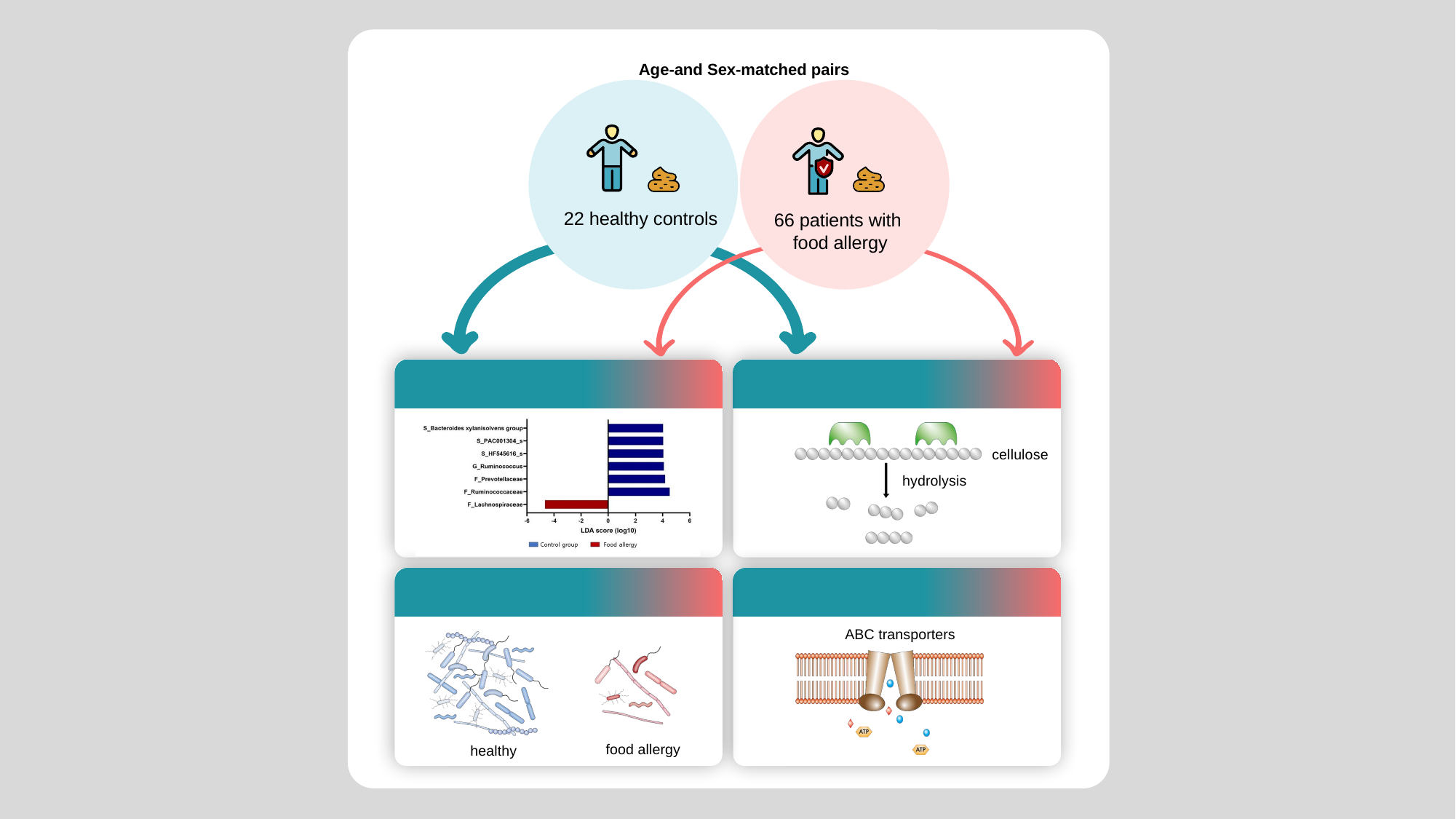

Age-and Sex-matched pairs
22 healthy controls
66 patients with
food allergy
Differential fecal microbiota
Dietary fiber breakdown &
generating short-chain fatty acids
cellulose
hydrolysis
Differential diversity
Protective barrier function
ABC transporters
food allergy
healthy
